# Taking a Campus-Wide View of Age-Friendly Practices in Higher Education

**DOI:** 10.1093/geroni/igab046.041

**Published:** 2021-12-17

**Authors:** Lauren Bowen, Nina Silverstein, Susan Whitbourne, Joann Montepare

**Affiliations:** 1 University of Massachusetts, Boston, University of Massachusetts Boston, Massachusetts, United States; 2 University of Massachusetts Boston, Boston, Massachusetts, United States; 3 Lasell University, Newton, Massachusetts, United States

## Abstract

The first AFU principle is to “encourage the participation of older adults in all the core activities of the university, including educational and research programs.” As this suggests, a crucial goal of age inclusivity in higher education is to resist the siloing of older adults and age-inclusive efforts in age-specific programs and cohorts. In response, the Age-Friendly Inventory and Campus Climate Survey (ICCS) assessment was designed to assess age-inclusivity across seven areas of institutional activity: outreach & engagement, personnel, physical environment, research, services & resources, student affairs, and teaching & learning. By restructuring and expanding the “pillars” of institutional activity outlined by AFU principles, the ICCS presents two key advantages for benchmarking AFU practices: (1) it traces age-inclusivity across many facets of institutional operations; and (2) it prompts participants and report readers to recognize their role in current and potential age-inclusive efforts, regardless of their role or department on campus.

